# Evaluating various properties of nanohydroxyapatite synthesized from eggshells and dual-doped with Si^4+^ and Zn^2+^: An in vitro study

**DOI:** 10.1016/j.heliyon.2024.e35907

**Published:** 2024-08-08

**Authors:** Shaza Alashi, Isam Alkhouri, Ibrahim Alghoraibi, Nabil Kochaji, Abdullah Houri, Mawia Karkoutly

**Affiliations:** a Department of Oral and Maxillofacial Surgery, Faculty of Dentistry, Damascus University, Damascus, Syrian Arab Republic; b Department of Physics, Faculty of Science, Damascus University, Damascus, Syrian Arab Republic; c Department of Oral Pathology, Faculty of Dentistry, Damascus University, Damascus, Syrian Arab Republic; d Department of Pediatric Dentistry, Faculty of Dentistry, Damascus University, Damascus, Syrian Arab Republic

**Keywords:** Nano-hydroxyapatite, Eggshells, Dopants, Biocompatibility

## Abstract

**Background:**

This study aimed to evaluate morphological, chemical and biocompatible properties of nanohydroxyapatite (N-HA) synthesized from eggshells and dual-doped with Si4+ and Zn2+.

**Methods:**

In the current study, N-HA was synthesized from chicken eggshells using the wet chemical precipitation method and doped with Si4+ and Zn2+. The physical assessment was carried out using field emission scanning electron microscopy (FE-SEM), energy dispersive X-ray (EDX) analysis, and X-ray diffraction (XRD) analysis. Crystal size was calculated using the Scherrer equation. Cytotoxicity was studied in vitro using the MTT (3-(4,5-Dimethylthiazol-2-yl)-2,5-Diphenyltetrazolium Bromide) cytotoxicity assay. The optical density (OD) of each well was obtained and recorded at 570 nm for 24 h (t1), 48 h (t2), 72 h (t3), and 5 days (t4) using a microplate reader.

**Results:**

The results of Si–Zn-doped HA showed a high specific surface area with an irregular nano-sized spherical particle structure. The atomic percentage provided the ratio of calcium to phosphate; for non-doped HA, the atomic Ca/P ratio was 1.6, but for Si–Zn-doped HA, where Zn+2 Ca and Si + replaced 4 substituted P, the atomic ratio (Ca + Zn)/(P + Si) was 1.76. The average crystal size of Si–Zn-doped HA was 46 nm, while for non-doped HA it was 61 nm. both samples were non-toxic and statistically significantly less viable than the control group After 5 days, the mean cell viability of Si–Zn-doped HA (79.17 ± 2.18) was higher than that of non-doped HA (76.26 ± 1.71) (P = 0.091).

**Conclusions:**

The MTT assay results showed that Si–Zn-doped HA is biocompatible. In addition, it showed characteristic physiochemical properties of a large surface area with interconnected porosity.

## 1 Background

Bone is a highly organized nanocomposite tissue composed of an organic part, mainly collagen type I, and a mineral part [1]. Biological apatite forms the mineral part of the bone, which consists of nano-sized platelet-shaped carbonated calcium phosphate particles located parallel to the long axis of collagen fibers [2].

Bone loss may occur due to multiple causes, such as periodontitis, jaw pathology, traumatic injuries, or after tooth extraction [3]. Bone grafting has become an ordinary procedure that includes the replacement of the missing part of the bone with a bone substitute that can trigger bone regeneration and convert it into bone tissue [4].

Due to their biocompatibility and ability to be resorbed by osteoclast cells, calcium phosphate compounds are considered one of the most commonly used synthetic bone graft materials [5]. In particular, hydroxyapatite (HA) with the chemical formula Ca_10_(OH)_2_(PO_4_)_6_ due to its biological properties and the similarity to the mineral part of bone [6]. Bone graft material should have the ability to dissolve over time and allow new bone to form instead. Since the degradation rate of HA is highly dependent on its particle size, nanoparticles of HA (N-HA) dissolve faster than conventional HA due to their high surface-to-volume ratio [7]. From a biological perspective, N-HA showed superior biocompatibility and osteointegration as a bone substitute material compared to porous HA in vivo studies. N-HA showed higher efficacy in new bone formation [8] due to the presence of nanofeatures on its surface, which alter the surface energy and increase affinity for extracellular protein adsorption and cell adhesion [9].

Many methods could be used to synthesize N-HA. Wet chemical precipitation is a simple and commercial method in which calcium and phosphorus precursors are mixed in an aqueous medium with a pH above 9 and constantly stirred over a certain period [10]. Although wet chemical precipitation is considered one of the earliest recognized aqueous phase synthesis methods of HA, a powder with different physical properties can be produced by changing the reaction factors, particularly pH, precursors, and aging time, which could modulate the biological properties of the synthesized N-HA [11].

Doping N-HA with foreign ions has piqued researchers' interest in recent years. The HA crystal lattice contains a variety of ionic substitutions, including carbonate and magnesium as important anionic and cationic elements in bone minerals [12]. Ion doping of N-HA affects both the morphology and solubility of N-HA, but the influence on cell behavior depends on the choice of the doping ions [13].

Trace elements are considered important in the nutritional and metabolic processes of the human body, especially for bone growth and development. Zinc (Zn^2+^) is an essential trace element in the precipitation and mineralization of the extracellular matrix [14]. In addition to its role as an anti-bacterial, zinc also increases the osteoblast response in vitro and in vivo studies [14]. Silicon (Si^4+^) is found in bones and connective tissue [15]. It can induce cellular activities such as osteogenic differentiation, which leads to improving the biological activity of N-HA. In addition, Si^4+^ has an angiogenic effect [16]. In vivo study with MG-63 osteoblast-like cells, Makarova et al. [17] demonstrated the favorable biological properties of co-substituted HA with Zn^2+^ and Si^4+^ compared to conventional HA.

Many researchers investigated the synthesis of HA from bioresources such as animal bones, coral, shells, and eggshells. The chicken eggshell consists of three layers: cuticle, spongia, and lamella. These layers contain a protein matrix bound to calcium carbonate crystals [18].

Chicken egg shells contain calcium, magnesium, iron, copper, and other mineral components, making them a material that can be used as a precursor for the synthesis of bone substitute materials [19]. Khattimani et al. [20] investigated eggshell-derived HA as a bone graft material for maxillary cystic bone defects. They found that the defects grafted with eggshell-derived HA showed complete bone formation, compared to non-grafted control defects that showed insignificant bone formation.

In the current study, N-HA was synthesized from chicken eggshells using the wet chemical precipitation method and doped with Si^4+^ and Zn^2+^ to obtain a material similar to natural bone apatite. This study aimed to evaluate morphological, chemical and biocompatible properties of nanohydroxyapatite (N-HA) synthesized from eggshells and dual-doped with Si4+ and Zn2+.

## 2 Materials and methods

### 2.1 Study design and ethics

This was an in vitro comparative experimental study. It was conducted at the Department of Oral and Maxillofacial Surgery, Faculty of Dentistry, Damascus University, between August 2023 and December 2023. Ethical approval was obtained from the Local Ethics Committee of Damascus University (N1592), and it was conducted in full accordance with CRIS Guidelines (Checklist for Reporting In-Vitro Studies) [21].

### 2.2 Synthesis of N-HA from chicken eggshells

An amount of hen's white eggshells was collected and cleaned using distilled water, then the eggshells were dried and heated at 1000^○C^ for 1 h, eliminating the organic compounds, releasing CO_2_, and leaving only calcium oxide CaO, as shown by the following reaction [22]:

$$eggshell\;\overset{\Delta }{\to }{CaCO}_{3\;}\overset{\Delta }{\to }CaO+{CO}_{2}$$

Then 1 mol (56 g) of CaO was hydrated with 2L of H_2_O and left to fully react on a magnetic stirrer at 1000 rpm and $20^{{}^{\circ}C}$ for 24 h, resulting in calcium hydroxide Ca(OH)_2_ as the following reaction:

$$CaO+H_{2}O\;\overset{\Delta }{\to }\;{Ca\left(OH\right)}_{2}$$

The required amount of 85 % orthophosphoric acid (H_3_PO_4_) was weighed using an analytical balance. 68.51 g of H_3_PO_4_ was added to the Ca(OH)_2_ solution at a rate of 1.5 mL/min while monitoring the pH of the solution to a pH-meter during acid addition. Stirring was carried out continuously at 1000 rpm and a temperature of 20^°C^ for 24 h to support the maturation of the mixture. 0.2 mol (7 g) of NH_4_OH was dropped onto the mixture as a buffer until the pH of the mixture was above 10. The precipitate was washed with distilled water and dried, then ground with a mortar and pestle. The sintering phase was carried out at 1200^°C^ for 1 h to achieve crystal growth [23,24].

### 2.3 Doping N-HA with Si^+4^ and Zn^+2^

The precursor of Zn^+**2**^ is aqueous zinc nitrate ZnNO_3_.6H_2_O (sigma Aldrich grade 98 %), while the precursor of Si^+4^ is tetraethyl orthosilicate -TEOS- (Sigma Aldrich grade 99 %). Both precursors were added to the calcium hydroxide Becher before the orthophosphoric acid was added. The chemical formula of the final production is [25,26]:

$${Ca}_{10-X}{Zn}_{X}{\left({PO}_{4}\right)}_{6-Y}{\left(SiO_{4}\right)}_{Y}{\left(OH\right)}_{2-Y}$$

The concentration of Zn and Si is 0.65 wt% and 0.85 wt%, respectively. with the consideration that if Zn content is higher than 1.2 wt%, this will result in cytotoxicity and a leak in substitution of zinc ions into HA crystal lattice [27]. Friederichs et al. [28] investigated the ionic co-substitution of zinc and silicon in hydroxyapatite, exploring different concentrations of these. They determined a (Ca + Zn)/(P + Si) ratio of 1.685 at Zn = 0.1 and Si = 0.3 wt%. The doping concentrations in this study were selected to achieve a Ca + Zn/P + Si ratio suitable for bone regenerative applications, which closely resembles the conventional Ca/P ratio in bone minerals. Two samples were obtained by the same synthetic steps mentioned previously; the first is Si–Zn-doped HA, and the second is non-doped HA.

### 2.4 FE-SEM characteristics

Field Emission Scanning Electron Microscopy (FE-SEM ZEISS Sigma 300, ZEISS, Baden-Württemberg, Germany) was used to study the microstructure and surface topography and estimate the particle sizes of both samples [29].

### 2.5 EDX analysis

Energy Dispersive X-ray analysis was done on both samples using FE-SEM, which is equipped with EDS (ZEISS Smart EDS, ZEISS, Baden-Württemberg, Germany) and EDX software to determine the elemental composition of the samples [29].

### 2.6 XRD analysis

X-ray Diffraction analysis of both samples was performed using a powder diffractometer system (STADI P, STOE Co., Illinois, United States) to study the crystallography structure, phase purity and physical properties of the samples. The patterns were obtained with CuКα radiation with λ = 0.15406 nm at 40 kV and 30 mA settings. XRD was recorded in a 2ϴ range of 20^°^–60^°^ with a step size of 0.02֯ and step duration of 0.5 s. Crystal size was calculated using the Scherrer equation [30]:

$$D=\frac{\kappa \lambda }{\beta \;\cos\;\theta }$$

D is crystal size, κ is Scherrer constant = 0.94, λ is the X-ray wavelength, β is the line broadening at FWHM in radians, and θ is the Braggs angel in degree [29].

### 2.7 Cell viability assay

The cytotoxicity of both samples was studied using the MTT (3-(4,5-Dimethylthiazol-2-yl)-2,5-Diphenyltetrazolium Bromide) assay and the MC3T3-E1 cell line as osteoblast-like cell precursors derived from mouse clavaria (99072810-1VL MC3T3-E1 MOUSE C57BL/6 CALVARIA, Sigma-Aldrich, Missouri, United States) [31]. Plain culture medium with 0 % extract was considered as a control medium [32]. The cells were seeded in 96-well plates (Multiscreen® 96 well Plate, Sigma-Aldrich, Missouri, United States) at a density of 104 cells per well and incubated in 100 μL of DMEM/F12, supplemented with 10 % heat-inactivated foetal bovine serum (FBS) (F7524 - Fetal Bovine Serum, Sigma-Aldrich, Missouri, United States) for 24 h. The culture media were replaced with fresh serum-free culture media containing serial dilutions of the sample, and the cells were incubated for 4 h. Then, the media were replaced with 100 μL of fresh, complete media for an additional 24 h. The medium was replaced with 100 μL of fresh medium containing MTT, giving a final MTT concentration of 0.5 mg/mL, and cells were further incubated in CO_2_ incubator (LEEC Culture Safe TOUCH CO2 Incubators, LEEC Ltd., Nottingham, United Kingdom) for 4 h at 37 °C. After 4 h, the medium was aspirated, and the MTT formazan generated by live cells was dissolved in 100 μL of DMSO (Dimethyl Sulfoxide – DMSO, Sigma-Aldrich, Missouri, United States). The optical density (OD) of each well was obtained and recorded at 570 nm for 24 h (t_1_), 48 h (t_2_), 72 h (t_3_), and 5 days (t_4_) using a microplate reader (UT-6550 Microplate Reader, MRC Ltd., Essex, United Kingdom). Cell viability for each sample was calculated using the following formula [33,34]:

$$Viab.\;\%=\frac{100\times {OD}_{s}}{{OD}_{c}}$$

OD_S_: is the mean value of measured optical density of the tested sample. OD_C_: is the mean value of measured optical density of the control sample, which is plain culture medium with 0 % extract.

### 2.8 Statistical analysis

Statistical analysis was performed using IBM SPSS software version 24 (IBM SPSS Statistics® version 24, IBM Corp., New York, USA). For the MTT assay data, the one-way ANOVA test was used to compare the three groups that were the Si–Zn-doped HA group, the non-doped Ha group, and the control group, followed by the post-hoc Tukey test for multiple comparisons. Descriptive statistics were expressed as mean and standard deviation. P ≤ 0.05 was considered statistically significant.

## 3 Results

### 3.1 FE-SEM characterization

**Fig. 1:**
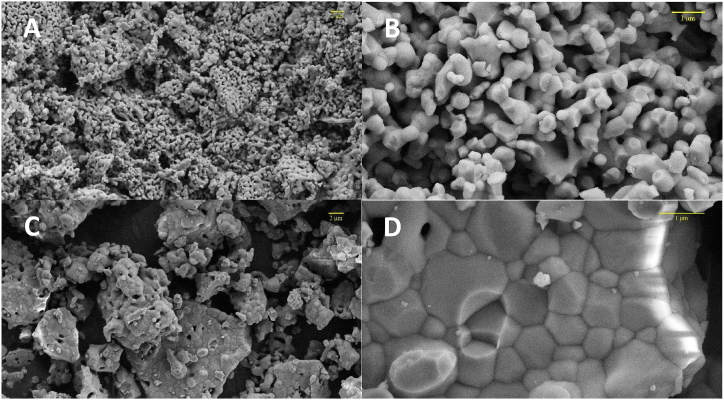
FE-SEM shows the microstructure of both doped and non-doped HA samples at magnifications of ×5000, and ×30000. **(A + B)** Si–Zn-doped HA revealed spherical particles and interconnected porosity. **(C + D)** non-doped HA presented high-agglomerated hexagonal particles.

**Fig. 2:**
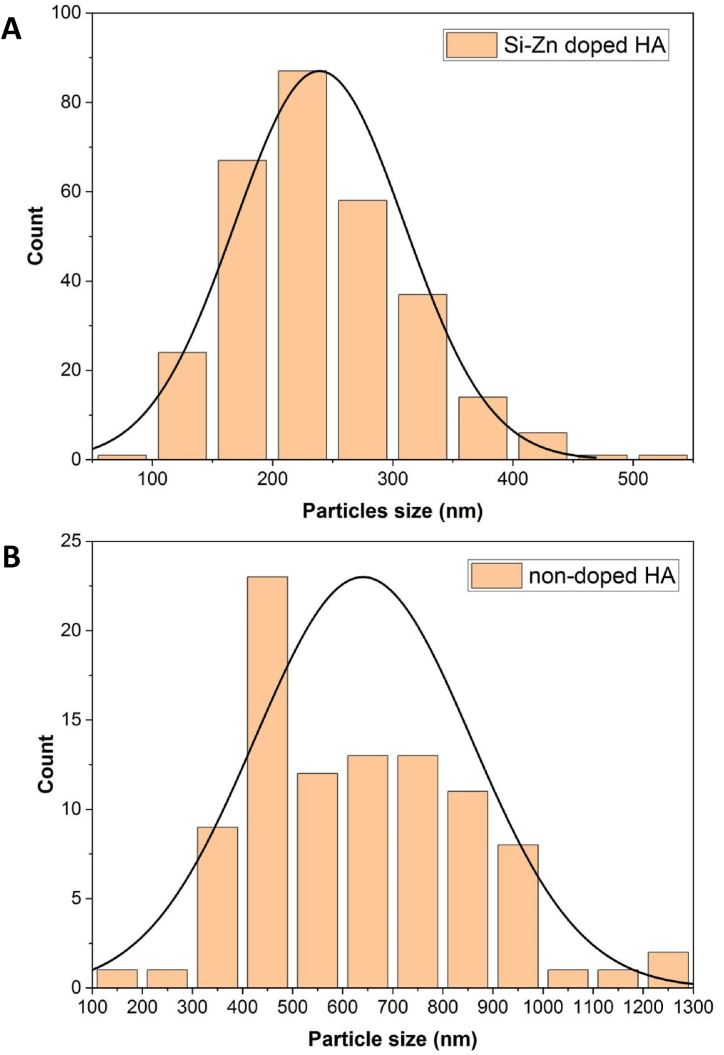
The particle size distribution of doped and non-doped HA. **(A)** doped HA. **(B)** non-doped HA. Si–Zn-doped HA has smaller particles than non-doped HA.

The results of Si–Zn-doped HA showed a high specific surface area with an irregular nano-sized spherical particle structure [Fig. 1(A and B)]. However, the results of non-doped HA showed highly agglomerated hexagonal particle clusters [Fig. 1(C and D)]. The particle diameters of doped HA ranged from 91 nm to 538 nm, and the average diameter was 239 nm, with an average porosity of 20 % [Fig. 2 (A)]. In contrast, non-doped HA had larger particles ranging from 190 nm to 1.2 μm in diameter and an average diameter of 640 nm [Fig. 2 (B)].

### 3.2 EDX analysis

**Fig. 3:**
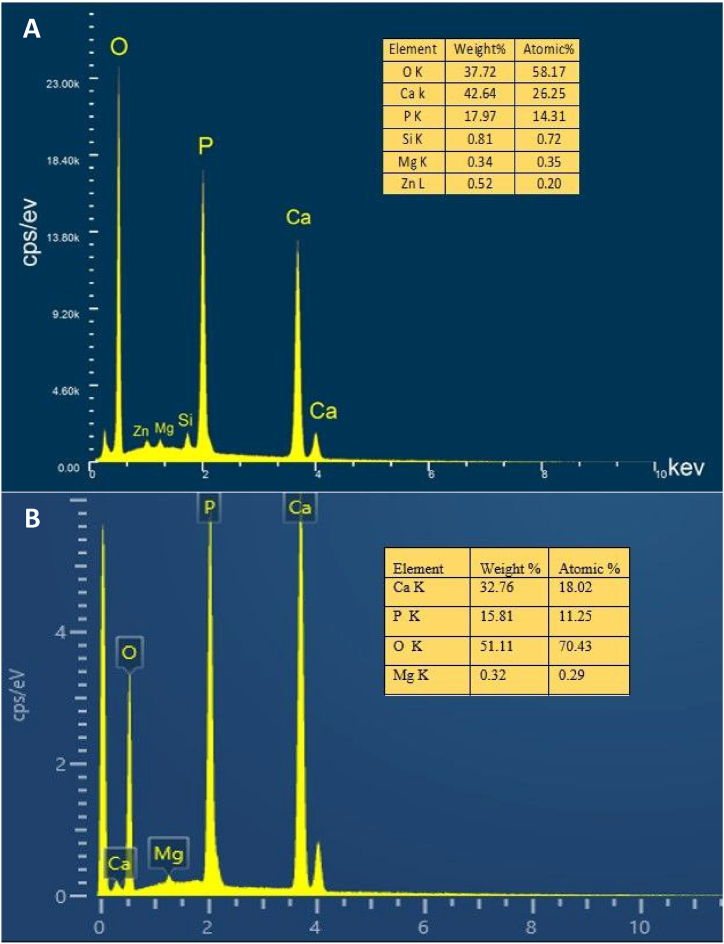
Typical EDX spectrum of both doped and non-doped HA with elements' weights and atomic percentages **(A)** doped HA. **(B)** non-doped HA. Peaks of Zn and Si are presented in doped HA, and a peak of Mg is presented in both samples.

The standard EDX spectra recorded from both samples are shown in Fig. 3(A and B). Characteristic peaks of Ca, P, O, and Mg are shown in both samples. However, peaks of Zn and Si are shown in the spectra of doped HA [Fig. 3 (A)]. The weight and atomic percentages of the element are shown in the tables in Fig. 3(A and B). The atomic percentage provided the ratio of calcium to phosphate; for non-doped HA [Fig. 3 (B)], the atomic Ca/P ratio was 1.6, which was very close to the Ca/P ratio of stoichiometric hydroxyapatite (1.67), but for Si–Zn-doped HA, where Zn+2 Ca and Si + replaced 4 substituted P, the atomic ratio (Ca + Zn)/(P + Si) was 1.76, representing a non-stoichiometric compound [Fig. 3 (A)].

### 3.3 XRD analysis

**Fig. 4:**
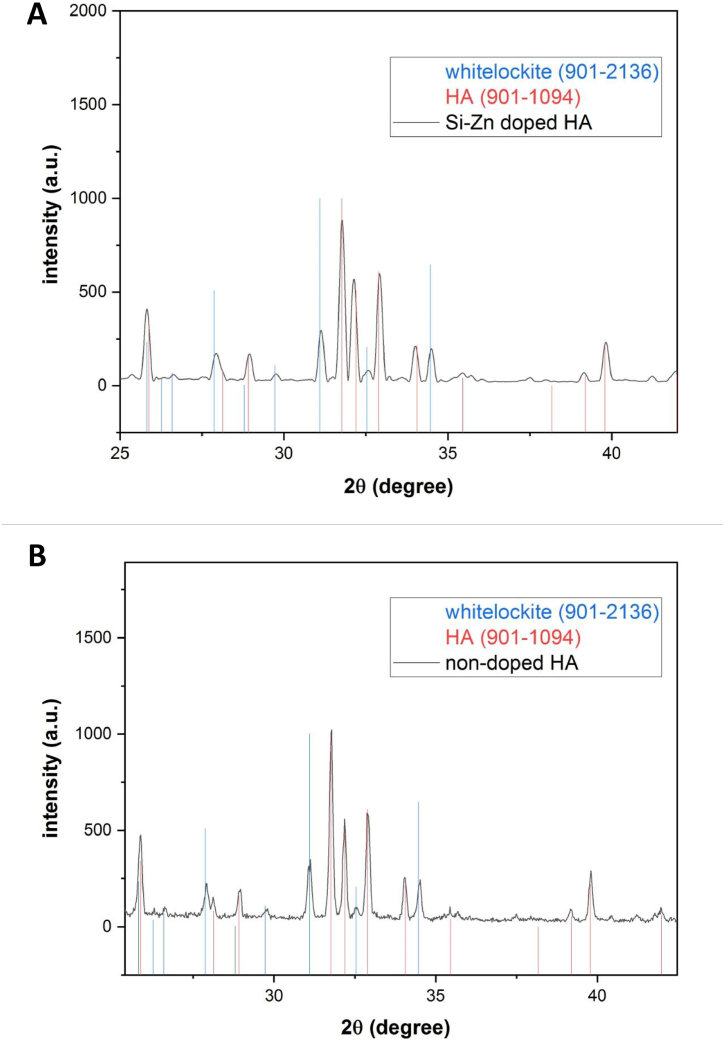
XRD patterns of both samples with references. **(A)** Doped HA. **(B)** non-doped HA. The Si–Zn-doped HA pattern showed peaks shifting to higher and lower 2ϴ as a result of doping HA with Zn and Si, respectively.

**Fig. 5:**
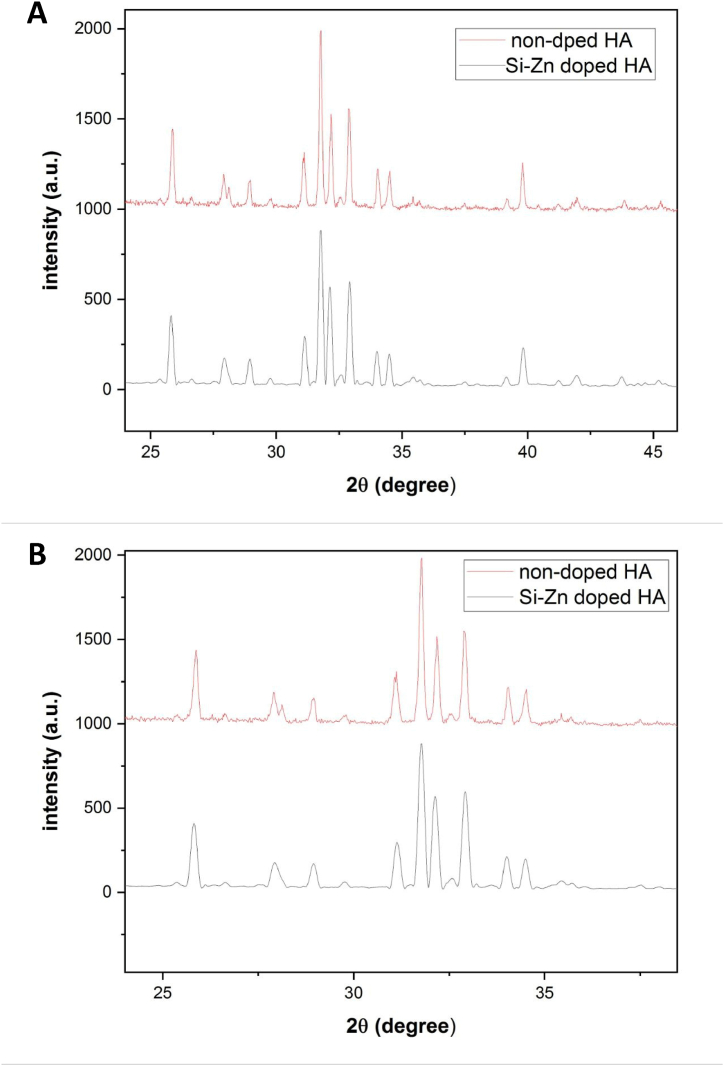
XRD patterns for doped and non-doped HA. Doped HA showed less peak intensity **(A)** and lower FWHM **(B)** than non-doped HA.

The XRD patterns of doped and non-doped HA are shown in Fig. 4(A and B). Both sample spectra agreed well with the reference pattern of hexagonal HA (JCPDS #901–1094) and the peaks of trigonal whitlockite (JCPDS #901–1094). 2136) were detected as a secondary phase. The peaks of Si–Zn-doped HA were shifted to higher and lower 2ϴ compared to the HA reference. These shifts can be attributed to the substitution of Zn and Si ions in the crystal lattice [Fig. 4 (A)]. The XRD pattern of non-doped HA showed higher intensity and sharper peaks compared to doped HA. This discrepancy suggests that doped HA had lower crystallinity than non-doped HA, even though both samples were sintered at the same temperature [Fig. 5(A and B)]. The Sherrer equation was used to calculate the crystal size of both samples. The average crystal size of Si–Zn-doped HA was 46 nm, while for non-doped HA it was 61 nm.

### 3.4 Cell viability

**Fig. 6:**
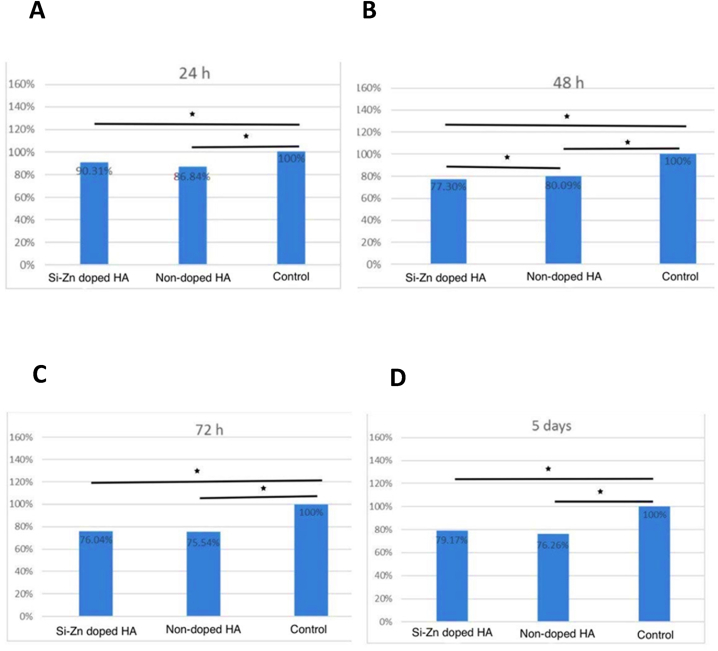
MTT assay results of MC3T3-E1 cells at t_1_, t_2_, t_3_, and t_4_. **(A)** t_1_. **(B)** t_2_. **(C)** t_3_. **(D)** t_4_. Both doped and non-doped HA samples showed viability during the 5 days of testing. Asterisks indicate statistically significant differences.

The cell viability of both samples was examined using the MTT assay. The results showed that both samples were non-toxic and statistically significantly less viable than the control group (Table 1) [Fig. 6(A–D)]. After 5 days, the mean cell viability of Si–Zn-doped HA (79.17 ± 2.18) was higher than that of non-doped HA (76.26 ± 1.71) (Table 1) [Fig. 6 (A)]. However, there was no statistically significant difference between these two groups at t_1_ (*P* = 0.587) [Fig. 6 (A)], t_3_ (*P* = 1.000) [Fig. 6 (C)], and t_4_ (*P* = 0.091) (Table 2) [Fig. 6 (D)]. There was a statistically significant difference at t_2_ (*P* < 0.05) (Table 2) [Fig. 6 (B)]. In addition, there was a statistical significant difference between the control group and doped HA group (p < 0.05), and the control group and non-doped HA (p < 0.05) at different time points.

**Table 1:** Results of a one-way ANOVA test for comparison between groups at different time points.

Time points	Groups	Mean	SD	*P*-value
t_1_	Si–Zn doped HA	90.31 %	2.22	0.001^a^
	Non-doped HA	86.84 %	5.67	
	Control	100 %	0.00	
t_2_	Si–Zn doped HA	77.30 %	1.43	<0.001^a^
	Non-doped HA	80.09 %	1.83	
	Control	100 %	0.00	
t_3_	Si–Zn doped HA	76.04 %	12.75	0.022^a^
	Non-doped HA	75.54 %	15.18	
	Control	100 %	0.00	
t_4_	Si–Zn doped HA	79.17 %	2.18	<0.001^a^
	Non-doped HA	76.26 %	1.71	
	Control	100 %	0.00	

a Significant difference at p < 0.05.

**Table 2:** Results of a post-hoc Tukey test for multiple comparisons.

Time points	Multiple comparisons		Mean Difference	P value
**t**_**1**_	Si–Zn doped HA	Non-doped HA	3.47 %	0.587
		Control	9.69%-	0.011^a^
	Non-doped HA	Control	13.16%-	0.011^a^
**t**_**2**_	Si–Zn doped HA	Non-doped HA	2.79%-	0.049^a^
		Control	22.70%-	<0.001^a^
	Non-doped HA	Control	19.91%-	<0.001^a^
**t**_**3**_	Si–Zn doped HA	Non-doped HA	0.49 %	1.0
		Control	23.97%-	0.048^a^
	Non-doped HA	Control	24.46%-	0.043^a^
**t**_**4**_	Si–Zn doped HA	Non-doped HA	2.91 %	0.091
		Control	20.83%-	<0.001^a^
	Non-doped HA	Control	23.74%-	<0.001^a^

a Significant difference at p < 0.05.

## 4 Discussion

Biomaterials science has evolved significantly in the last few decades. HA caught the attention of researchers due to its similarity to bone biological apatite. Many methods can be used to synthesize HA. In the living body, biological mineralization occurs in body fluids to form nanoparticles of non-stoichiometric calcium phosphate substituted with several ions, particularly CO3^2−^ and Mg^2+^ [35]. It leads to the use of wet chemical precipitation for N-HA synthesis, especially from biowaste resources. N-HA synthesized from biowaste materials showed better biocompatibility when implanted in living tissues due to its homogeneity with bone tissue, both in physical and chemical characteristics [36]. Goloshchapov et al. [37] synthesized nanocrystalline hydroxyapatite from hen's shells utilizing wet precipitation. After annealing at 900○c, the particles reached a size of more than 150 nm and presented in cylindrical shape. Pon-On et al. [38] synthesized flat-plate-like particles of HA using fish as a precursor. The shape and size of the particles varied depending on the synthesis method using wet chemical precipitation in the synthesis of N-HA, which was associated with producing powder with low crystallinity and weak mechanical properties [10]. The sintering step was carried out to overcome this problem. The temperature used during the thermal processing of the N-HA powder stimulates the crystallization process within the particles. It is considered a crucial factor in determining the shape and size of particles [39]. The high temperature during sintering stimulates crystal growth, increases the particle size, and reduces the porosity of the powder [40]. The FE-SEM result of the non-doped HA sample showed large hexagonal particles with poor porosity after the powder was sintered at 1200^○^C. Swain et al. [39] got similar results as they demonstrated that when HA powder was sintered to 1200^○^C, 1250^○^c, and 1300^○^c, particle size increased with the increase in sintering temperature, and HA microstructure exhibited much less porosity. In Si–Zn-doped HA sample, the size of particle decreased and showed different shapes with high interconnect porosity even though the sample was sintered at the same high temperature (1200^○^C). This attribute suggested that doping N-HA with Zn^2+^ and Si^4+^ resulted in shrinkage of the crystal lattice due to the differences in ionic radius between dopant ions and substituted ions [41], or the doping process played an obstructing role in crystallization. This result is consistent with S. V. Makarova et al. study [17], which showed a decrease in the crystal lattice size of HA after it was dual-doped with Zn and Si. In the hexagonal crystal system of HA, Zn^2+^ ions substitute Ca^2+^ ions in the second position, and Si^4+^ ions substitute P^5+^ ions [42]. It causes changes in the lattice parameters and leads to changes in crystal size and crystallinity, which can be demonstrated by XRD results. The crystal size of the Si–Zn-doped HA sample (46 nm) was smaller than the crystal size of the non-doped HA sample (61 nm). Furthermore, peak shifts were observed in the XRD pattern of the Si–Zn-doped HA sample, which together with the EDX results, demonstrate the successful substitution of Zn^2+^ and Si^4+^ in the crystal structure and not only adsorption on its surface. When Zn^2+^ substitutes Ca^2^+, the peak shifts to a higher 2ϴ because Zn^2^+ has a smaller ionic radius. Likewise, Si4+ has a larger ionic radius than P^5+^ and the peak shifts to a lower 2ϴ. This observation was consistent with the Ofudje et al. [41] study, which studied the effect of doping Zn in different concentrations into HA and assumed that doping Zn into HA caused a decrease in Crystal size. Friederichs et al. [28] suggested that the dual doping of Zn and Si into HA led to defects in lattice parameters and the shrinking of HA crystal size. The incorporation of doping ions into the crystal lattice is considered an important factor for the biological behavior of the powder. This incorporation results in a small but continuous release of these ions during the bone remodeling process [43]. Dopant ions also influence the thermal stability of the synthesized N-HA and lead to decomposition into other phases even after sintering at high temperatures. The phase purity of both samples was examined using XRD. The results of doped and non-doped HA showed the presence of a second phase of whitlockite (WH) with the chemical formula Ca_18_Mg_2_(HPO_4_)_2_(PO_4_)_12_. WH is a calcium orthophosphate crystal that forms the second most abundant inorganic compound in bone [44]. WH has biological importance due to its biocompatibility, biodegradability, and negative surface charge [44]. WH has the same crystal structure as β-TCP but with the presence of magnesium at two of the calcium positions in the rhombohedral crystal lattice [45]. The WH phase appeared due to the use of chicken egg shells as a precursor in the synthesis since dry chicken eggshells contain about 3 % of their weight in magnesium [46]. Goloshchapov et al. [37] synthesized HA from hen shells, and the XRD analysis showed the presence of whitlockite as a second phase with the HA phase. Otherwise, Makarova et al. [17] demonstrated the formation of only one crystal phase (HA phase) and the appearance of an amorphous phase, which is visible with the increase in the concentration of dopants. The differences in phase purity can be explained by the variation of the precursors used in the synthesis of HA. From a biological perspective, the microstructure and surface topography of N-HA play an essential role in cell interaction. Lebre et al. [47] found that HA with spherical nanoparticles improved successful tissue remodeling compared to needle-like particles in both in vitro and in vivo studies. Bone regeneration scaffolds should have good porosity to promote bone ingrowth and cell function, adsorption of extracellular proteins, and support angiogenesis [48]. Therefore, for the Si–Zn-doped HA sample, the interconnected porosity and difference in pore size are considered suitable for cell adhesion and angiogenesis through the scaffold when implanted into living bone.

Alkaline phosphatases are Zn^2+^ metalized glycoproteins that play an important role in the maturation of newly formed bone by catalyzing the hydrolysis of phosphomonoester to inorganic phosphate. Essentially, their role is to create an alkaline environment that promotes the precipitation and mineralization of this inorganic phosphate on the extracellular matrix [49]. Doping N-HA with Zn^2+^ ensures the release of Zn^2+^ during the bone remodeling process, which increases the osteoblastic response to form new bone and, in addition, the antibacterial activity of zinc [41]. Silicon is considered an essential element for collagen synthesis in bones and other connective tissues [15]. Silicon has a stimulating effect on the proliferation and differentiation of osteoblast-like cells [16]. By inducing VEGF (Vascular Endothelial Growth Factor) expression, silicon promotes angiogenesis, which is considered crucial for bone ingrowth [15]. Hing et al. [50] tested the different concentrations of Si-doped into HA and implanted in the femoral intercondylar of New Zealand White rabbits. The optimal bone formation was found in the 0.8 wt% Si group.

In this study, in vitro analysis results of osteoblast-like cells (MC3T3-E1) showed that the doped sample (Si–Zn-doped HA) has higher cell viability than non-doped HA. Our results support the conclusion of Makarova et al. [17] showed that doping N-HA with Zn and Si improved biocompatibility, reduce cytotoxicity and increase the solubility of the material. The main limitation of this study was the use of only one concentration of Zn and Si.

## 5 Conclusions

N-HA can be synthesized from biowaste and doped with 0.65 wt% of Zn and 0.85 wt % of Si using a simple and inexpensive method. The final product showed characteristic physiochemical properties of a large surface area with interconnected porosity. The MTT assay results showed that N-HA doped with silicon and zinc is biocompatible.

## Ethics approval and consent to participate

This study was reviewed and approved by The Local Ethics Committee of Damascus University with the approval number: N1592, dated November 21, 2020.

## Consent for publication

Not applicable.

## Funding

This research is funded by 10.13039/501100020595Damascus University – funder No. 501100020595.

## Preprint

The manuscript has been previously published as a preprint on research square [51].

## Data availability statement

Data will be made available on request.

## CRediT authorship contribution statement

**Shaza Alashi:** Writing – original draft, Methodology, Investigation, Data curation, Conceptualization. **Isam Alkhouri:** Supervision, Project administration, Conceptualization. **Ibrahim Alghoraibi:** Project administration. **Nabil Kochaji:** Project administration. **Abdullah Houri:** Data curation. **Mawia Karkoutly:** Writing – review & editing.

## Declaration of competing interest

The authors declare that they have no known competing financial interests or personal relationships that could have appeared to influence the work reported in this paper.

## List of abbreviations

HA: Hydroxyapatite
N-HA: nanoparticles of hydeoxyapatite
Zn^2+^: Zinc
Si^4+^: Silicon
FE-SEM: field emission scanning electron microscopy
EDX: energy dispersive X-ray
XRD: X-ray diffraction
